# Anti-tumor effects of rivoceranib against canine melanoma and mammary gland tumour in vitro and in vivo mouse xenograft models

**DOI:** 10.1186/s12917-021-03026-1

**Published:** 2021-10-26

**Authors:** Qiang Li, You-Seok Kim, Ju-Hyun An, Jin-Ah Kwon, Sang-Hyun Han, Woo-Jin Song, Hwa-Young Youn

**Affiliations:** 1 Department of Veterinary Medicine, College of Agriculture, YanBian University, YanJi, JiLin China; 2 Laboratory of Veterinary Internal Medicine, Department of Veterinary Clinical Science, College of Veterinary Medicine, Seoul National University, Seoul, 08826 Republic of Korea; 3 KPC Corporation, Oporo, Opo-eup, Gwangju-si, Gyeonggi-do Korea; 4 HLB LifeScience Co., Ltd., Teheran-ro, Gangnam-gu, Seoul, Republic of Korea; 5 Department of Veterinary Internal Medicine and Research Institute of Veterinary Science, College of Veterinary Medicine, Jeju National University, Jeju, 63243 Republic of Korea

**Keywords:** Dog, Mammary gland tumour, Melanoma, Rivoceranib, VEGFR2

## Abstract

**Background:**

Rivoceranib, a novel tyrosine kinase inhibitor, exhibits anti-tumour effects by selectively blocking vascular endothelial growth factor receptor-2 (VEGFR2) in cancer cells. Recently, the therapeutic effects of rivoceranib on solid tumours have been elucidated in human patients. However, the anti-tumour effects of rivoceranib against canine cancer remain unclear. Here, we investigated the anti-tumour effects of rivoceranib using in vitro and in vivo mouse xenograft models.

**Methods:**

We performed cell proliferation, cell cycle, and migration assays to determine the effects of rivoceranib on canine solid tumour cell lines in vitro. Furthermore, apoptosis and angiogenesis in tumour tissues were examined using a TUNEL assay and immunohistochemistry methods with an anti-cluster of differentiation-31 antibody, respectively. Additionally, the expression levels of cyclin-D1 and VEGFR2 activity were determined using western blot analysis.

**Results:**

Rivoceranib treatment showed anti-proliferative effects and mediated cell cycle arrest in the canine melanoma cell line (LMeC) and the mammary gland tumour (MGT) cell line (CHMp). In animal experiments, rivoceranib decreased the average volume of LMeC cells compared to that following control treatment, and similar results were observed in CHMp cells. Histologically, rivoceranib induced apoptosis and exerted an anti-angiogenic effect in tumour tissues. It also downregulated the expression of cyclin-D1 and inhibited VEGFR2 activity.

**Conclusion:**

Our results show that rivoceranib inhibits proliferation and migration of tumour cells. These findings support the potential application of rivoceranib as a novel chemotherapeutic strategy for canine melanoma and MGTs.

**Supplementary Information:**

The online version contains supplementary material available at 10.1186/s12917-021-03026-1.

## Background

Cancer is one of the leading causes of death in dogs, and more than 50% of dogs older than 10 years of age develop at least one malignant tumour [1]. One of the most common malignant tumours in female dogs is the mammary gland tumour (MGT) [2]. In addition, a relatively common cancer in dogs is melanoma, which can occur in the oral cavity, eye, mucocutaneous junction, nail bed, foot pad, or gastrointestinal tract [3, 4]. Conventional alkylating agents for adjuvant chemotherapy (pulse or metronomic) have been used for MGT or melanoma dogs [5, 6]. Furthermore, recently, recombinant tyrosine kinase inhibitors such as toceranib and masitinib have been prescribed for clinical trials for dogs with solid tumours [1, 7, 8]. However, further clinical studies with large population, and research studies demonstrating their underlying mechanisms are needed.

Angiogenesis has been identified to play a crucial role in solid tumour malignancies and is essential for tumour proliferation, survival, and metastasis [8–10]. The vascular endothelial growth factor (VEGF) family and its receptors (VEGFRs) are considered to comprise the core components in tumour angiogenesis-related molecular mechanisms [11]. Recently, the possibility of inhibiting tumour cell signals arising from the activation of VEGFR2 has been demonstrated through several pharmacodynamic approaches, including receptor blockade (ramucirumab), seizure of the ligand (bevacizumab), and use of small-molecule inhibitors (sorafenib, sunitinib, apatinib, cediranib, and telatinib) [12].

Rivoceranib (also known as apatinib), a novel oral small-molecule selective tyrosine kinase inhibitor of VEGFR2, blocks endothelial and tumour cell proliferation and migration, thus inhibiting tumour growth [13, 14]. Previous studies have demonstrated its improved therapeutic efficacy against various types of carcinoma in humans [15–17]. Furthermore, our group previously reported that rivoceranib showed in vitro anti-tumor activity in canine MGT (CIPp and CIPm; derived from primary site and metastatic lymph node, respectively, with mammary adenocarcinoma) cell lines [18]. However, it is unknown whether rivoceranib plays a similar role in canine melanoma (LMeC; derived from metastatic lymph node with oral mucosa melanoma) and MGT (CHMp; derived from primary site with inflammatory mammary adenocarcinoma) cell lines [19, 20].

In this study, we aimed to investigate the anti-tumour effects of rivoceranib in in vitro and in vivo mouse xenograft models of canine melanoma (LMeC) and MGT (CHMp). The results provide novel insights into the inhibition of VEGFR2 by rivoceranib in canine cancer cells.

## Results

### Rivoceranib inhibits the proliferation and migration of canine melanoma and MGT cell lines in vitro

**Fig. 1:**
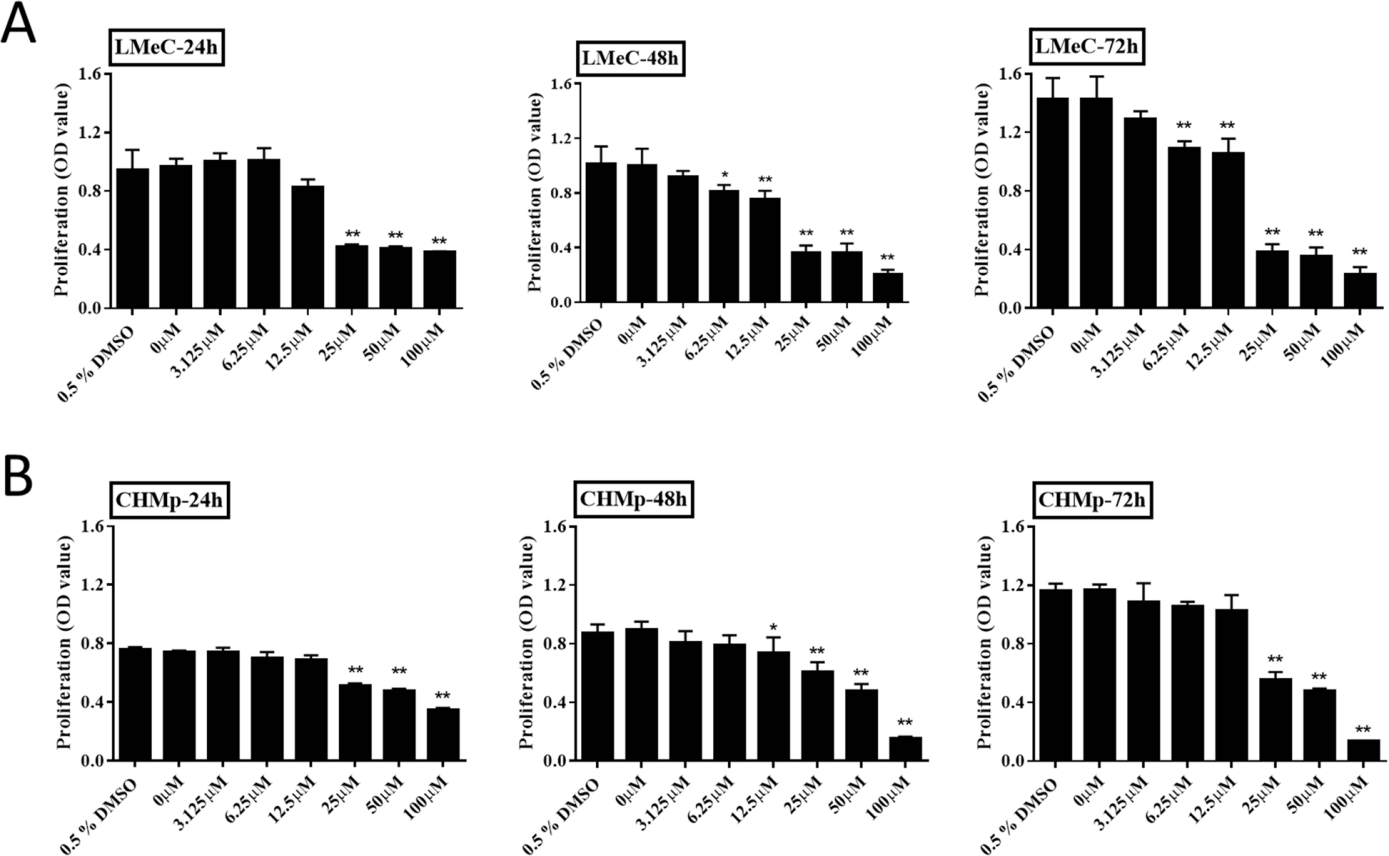
Effects of rivoceranib on the proliferation of canine solid tumour cell lines in vitro. **A**, **B** Viability of LMeC and CHMp cells after treatment with different concentrations of rivoceranib at 24, 48, and 72 h of treatment. All experiments were performed in triplicate. The data represent the mean ± standard deviation obtained from three independent experiments. * *p* < 0.05, ** *p* < 0.01 versus the control group (0.5% dimethyl sulfoxide) at the same time point

To assess the effects of rivoceranib on the proliferation of LMeC and CHMp cells, they were treated with different concentrations of the anti-cancer drug for 24, 48, and 72 h. Results of the cell counting kit (CCK)-8 assay showed that rivoceranib elicited inhibitory effects in a dose-dependent manner in both cell lines (Fig. 1A, B). Cell proliferation was significantly reduced at 25 μM after 24, 48, and 72 h of treatment compared to that in the control groups of both cell lines. Cell proliferation was also significantly reduced following treatment with 6.25 μM (LMeC) and 12.5 μM (CHMp) of rivoceranib after 48 h compared to that in the control group. However, no significant reduction in cell proliferation was observed at 3.125 μM (LMeC) and 12.5 μM (CHMp) of rivoceranib after 72 h. Therefore, for subsequent experiments, we treated the cells with 0, 12.5, and 25 μM of rivoceranib for 48 h.

### Rivoceranib promotes cell cycle arrest in canine tumour cell lines in vitro

**Fig. 2:**
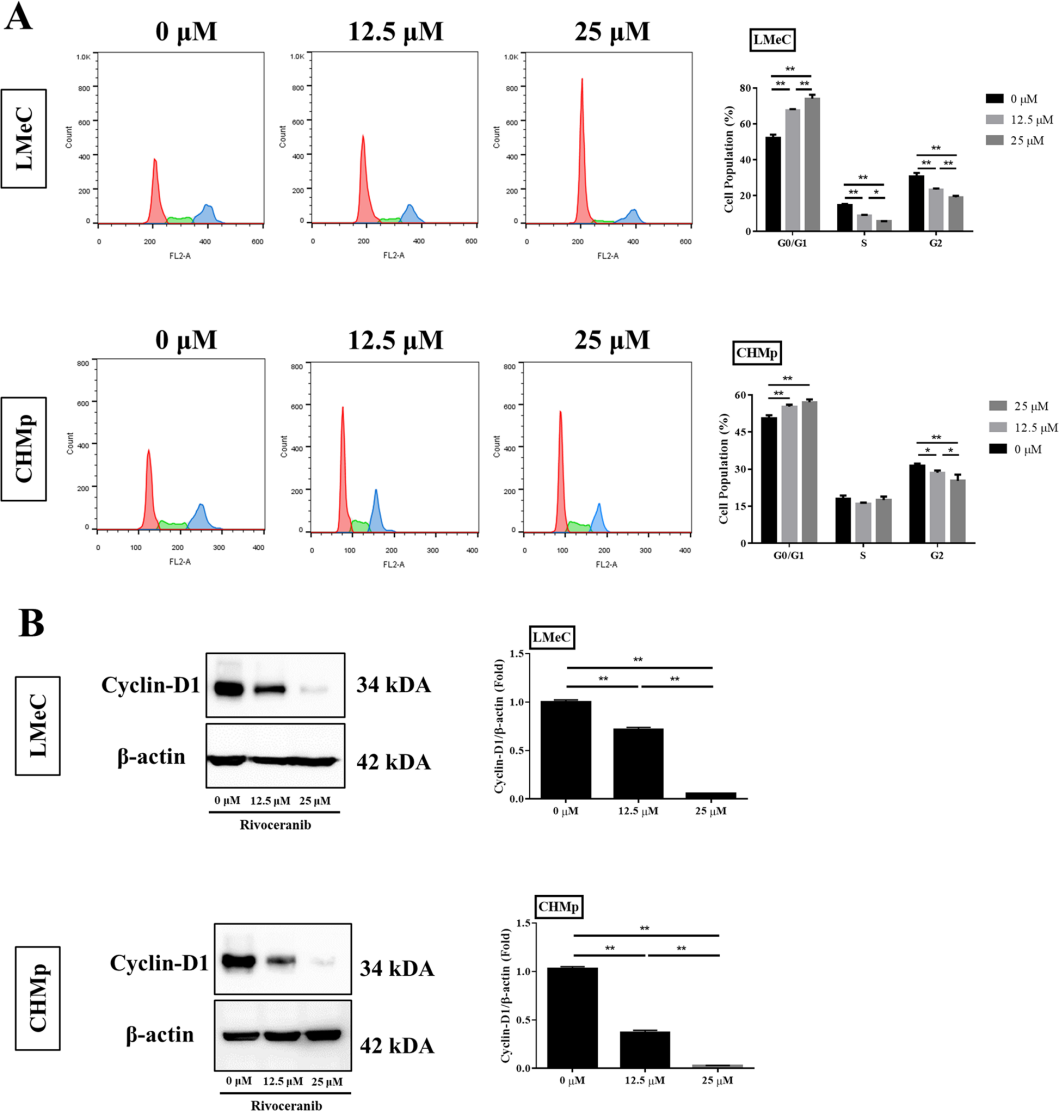
Effects of rivoceranib on cell cycle arrest in vitro. **A** Cell cycle distribution of LMeC and CHMp cells treated with rivoceranib was analysed by flow cytometry at 48 h. **B** The expression levels of cyclin-D1 were measured using western blot analysis. The intensity of the band on the films is presented as the relative ratio of cyclin-D1 to the band intensity of β-actin. Cropped blots are displayed. Samples were derived from same experiment, and blots were processed in parallel. The data represent the mean ± standard deviation obtained from three independent experiments. * *p* < 0.05, ** *p* < 0.01

The effect of rivoceranib on the cell cycle progression of LMeC and CHMp cells was investigated using fluorescence-activated cell sorting (FACS). The cells were treated with different concentrations (0, 12.5, and 25 μM) of rivoceranib for 48 h. These cells were harvested and analysed to determine their distribution among the G0/G1, S, and G2/M phases of the cell cycle. As shown in Fig. 2A, the G0/G1 ratio in the rivoceranib-treated groups was significantly higher than that in the control group (0 μM) in both cell lines, whereas the distribution of cells in the G2/M phase was significantly reduced in both the 12.5-and 25 μM-treated groups compared to that in the untreated control group. In addition, the S phase distribution of LMeC cells was significantly reduced in both the 12.5-and 25 μM-treated groups compared to that in the control group. In contrast, the S phase distributions of CHMp cells were similar in the rivoceranib-treated and untreated control groups.

The proteins extracted from the collected LMeC and CHMp cells were analysed by western blotting. A comparison of relative band intensities confirmed that the expression of cyclin-D1 was significantly lower in the rivoceranib-treated groups than in the untreated control group, and the difference between the 12.5-and 25 μM-treated groups was also significant in both cell lines (Fig. 2B). These results suggest that rivoceranib induces G0/G1 cell cycle arrest through the downregulation of cyclin-D1, thereby inhibiting cell cycle progression in LMeC and CHMp cells.

### Rivoceranib inhibits migration and induces apoptosis in canine tumour cell lines in vitro

**Fig. 3:**
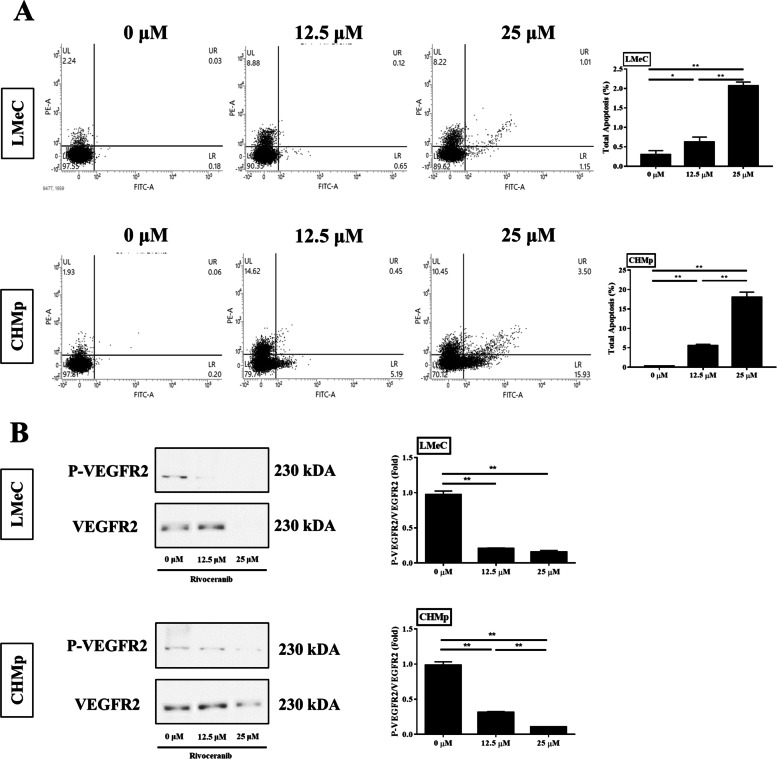
Effects of rivoceranib on the migration of canine solid tumour cell lines in vitro. **A** Cell migration abilities of LMeC and CHMp were measured at 0, 12, and 24 h of rivoceranib treatment. **B** Histograms showing the relative gap size of the control group at 0 h after rivoceranib treatment. The data represent the mean ± standard deviation obtained from three independent experiments. * *p* < 0.05, ** *p* < 0.01

The effect of rivoceranib on the migration abilities of LMeC and CHMp cells was evaluated using a wound-healing assay. As shown in Fig. 3, migration was significantly reduced in cells treated with rivoceranib in a concentration-dependent manner in both cell lines. These results showed the ability of rivoceranib to inhibit the mobility of canine tumour cell lines in vitro.

**Fig. 4:**
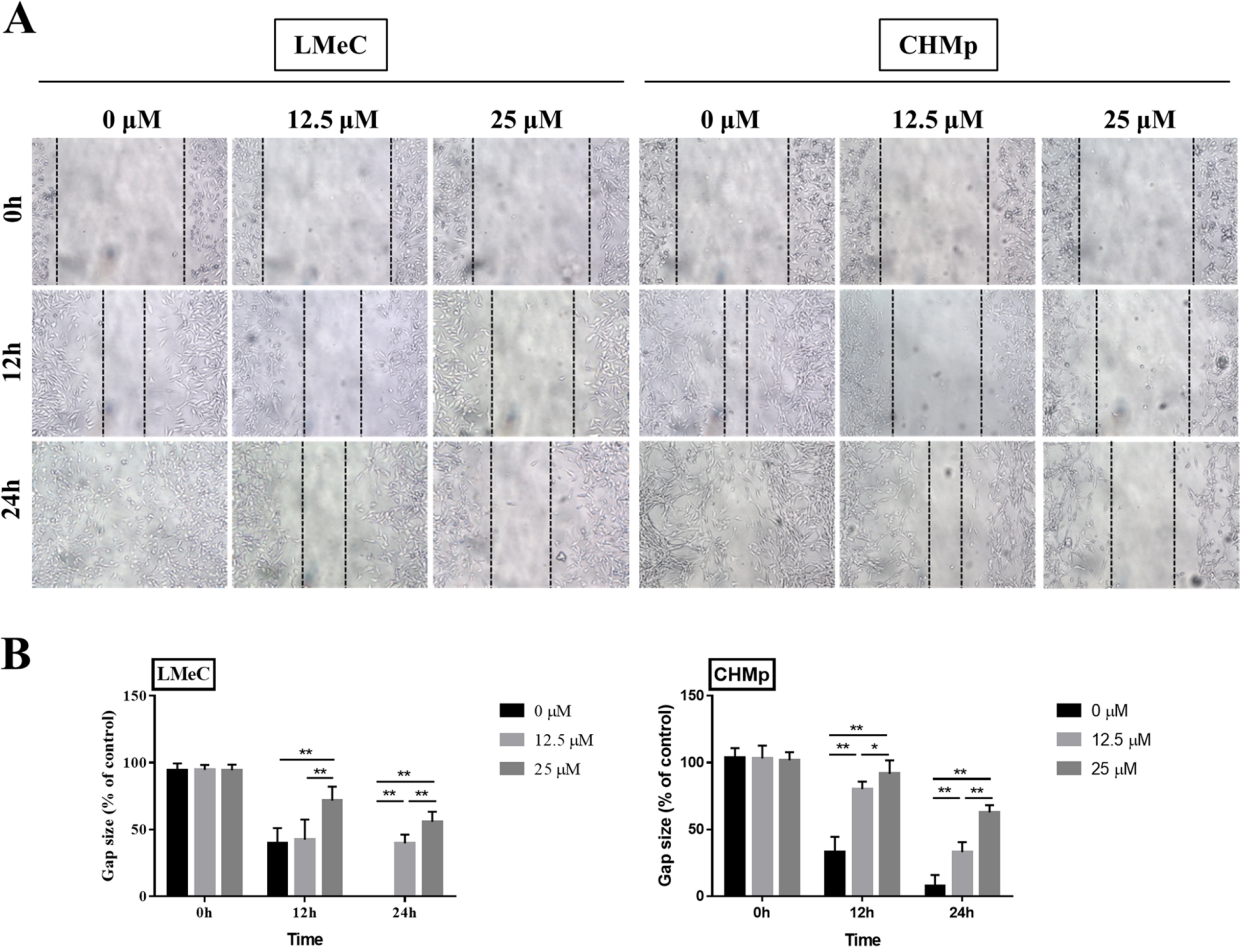
Effects of rivoceranib on the apoptosis of canine solid tumour cell lines. The percentage of apoptotic cells was measured by flow cytometry. Total apoptotic cells were quantified using Annexin V (FITC) and propidium iodide (PI) dual staining. Annexin V^+^/PI^−^ cells were considered to reflect early apoptosis, and Annexin V^+^/PI^+^ cells were considered to reflect late apoptosis. The data represent the mean ± standard deviation obtained from three independent experiments. * *p* < 0.05, ** *p* < 0.01

To evaluate apoptosis, LMeC and CHMp cells treated with rivoceranib for 48 h were harvested and analysed by FACS after Annexin V/ propidium iodide (PI) dual staining. The percentage of apoptotic cells was significantly increased in the cells treated with 12.5 and 25 μM rivoceranib compared to that in the untreated control group in both cell lines (Fig. 4). These results showed that rivoceranib also induces apoptosis in canine tumour cell lines in vitro.

### Rivoceranib suppresses tumour growth in xenograft mouse models

**Fig. 5:**
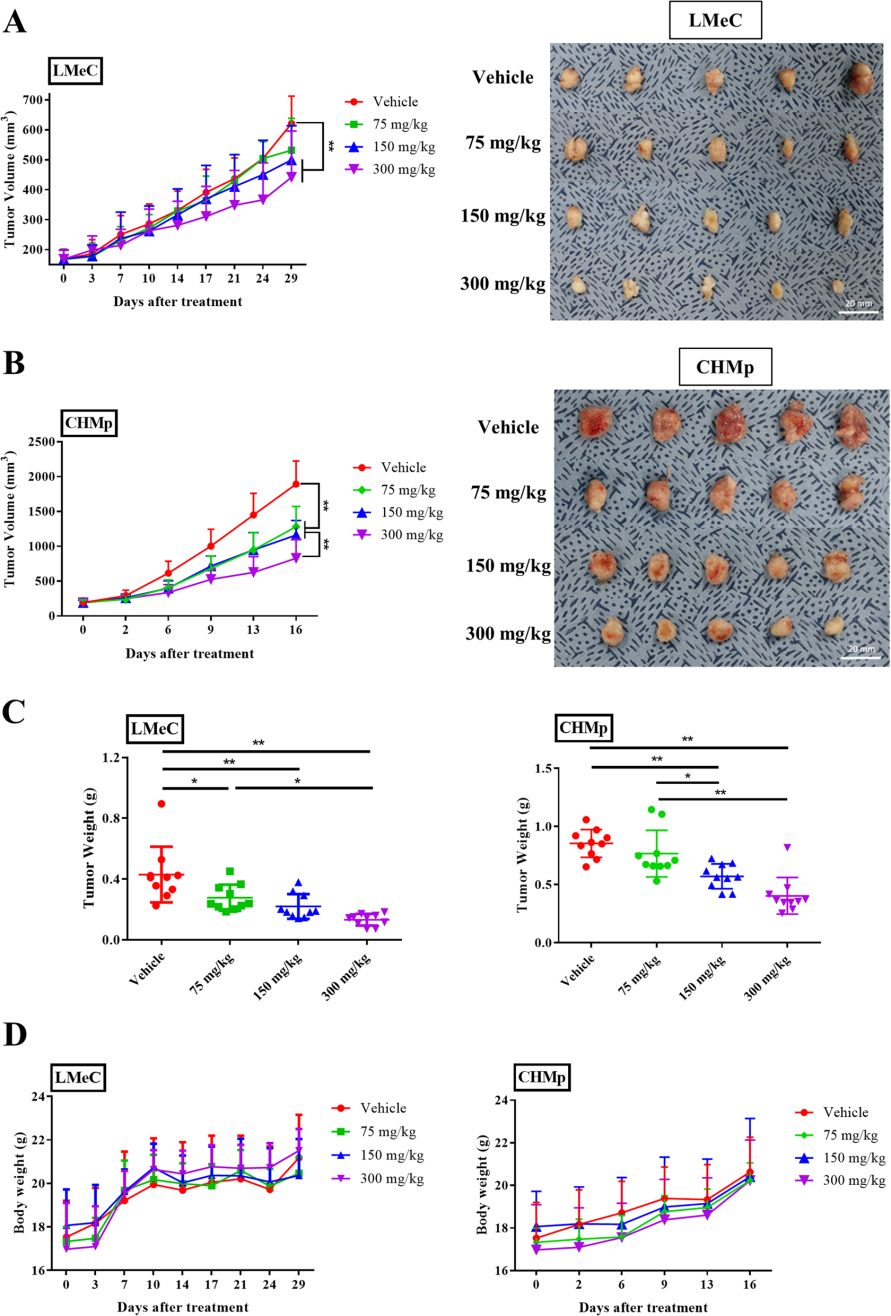
In vivo effects of rivoceranib in the xenograft model. **A** The mean LMeC tumour volumes in the four groups (vehicle and 75 mg/kg, 150 mg/kg, and 300 mg/kg rivoceranib-treated; *n* = 10 in each group), and images of the collected tumours from the xenograft models after 29 days of treatment. **B** The mean CHMp tumour volumes in the four groups (vehicle and 75 mg/kg, 150 mg/kg, and 300 mg/kg rivoceranib-treated; *n* = 10 in each group), and images of the collected tumours from the xenograft models after 16 days of treatment. **C** The tumour weights and **D** body weights of mice were monitored twice a week during rivoceranib treatment in both cell lines. The data represent the mean ± standard deviation * *p* < 0.05, ** *p* < 0.01

To further determine the anti-tumour activity of rivoceranib in vivo, LMeC and CHMp cell-xenografted mice were orally administered various concentrations of rivoceranib daily. The tumour volume in the control group (vehicle) rapidly increased compared to that in the treated groups. In the LMeC cell line, after 29 days of treatment, the tumour growth curve showed that there was a significant reduction in the tumour volume in the rivoceranib (150 and 300 mg/kg)-treated groups compared to that in the control group (Fig. 5A). In the CHMp cell line, after 16 days of treatment, the 150 and 300 mg/kg rivoceranib-treated groups exhibited a more significant inhibition of tumour volume growth (Fig. 5B) than the control group.

Furthermore, tumour weight was also measured after the mice were sacrificed; the results showed that the tumour weight significantly decreased in the mice treated with rivoceranib in a dose-dependent manner (Fig. 5C) in both cell lines. To detect severe side effects of rivoceranib in mouse models, body weight was also measured. The body weights of mice in the treatment and control groups were similar, with no significant differences observed (Fig. 5D) in both cell lines.

### Rivoceranib downregulates VEGFR2 phosphorylation and cyclin-D1 expression in vivo

**Fig. 6:**
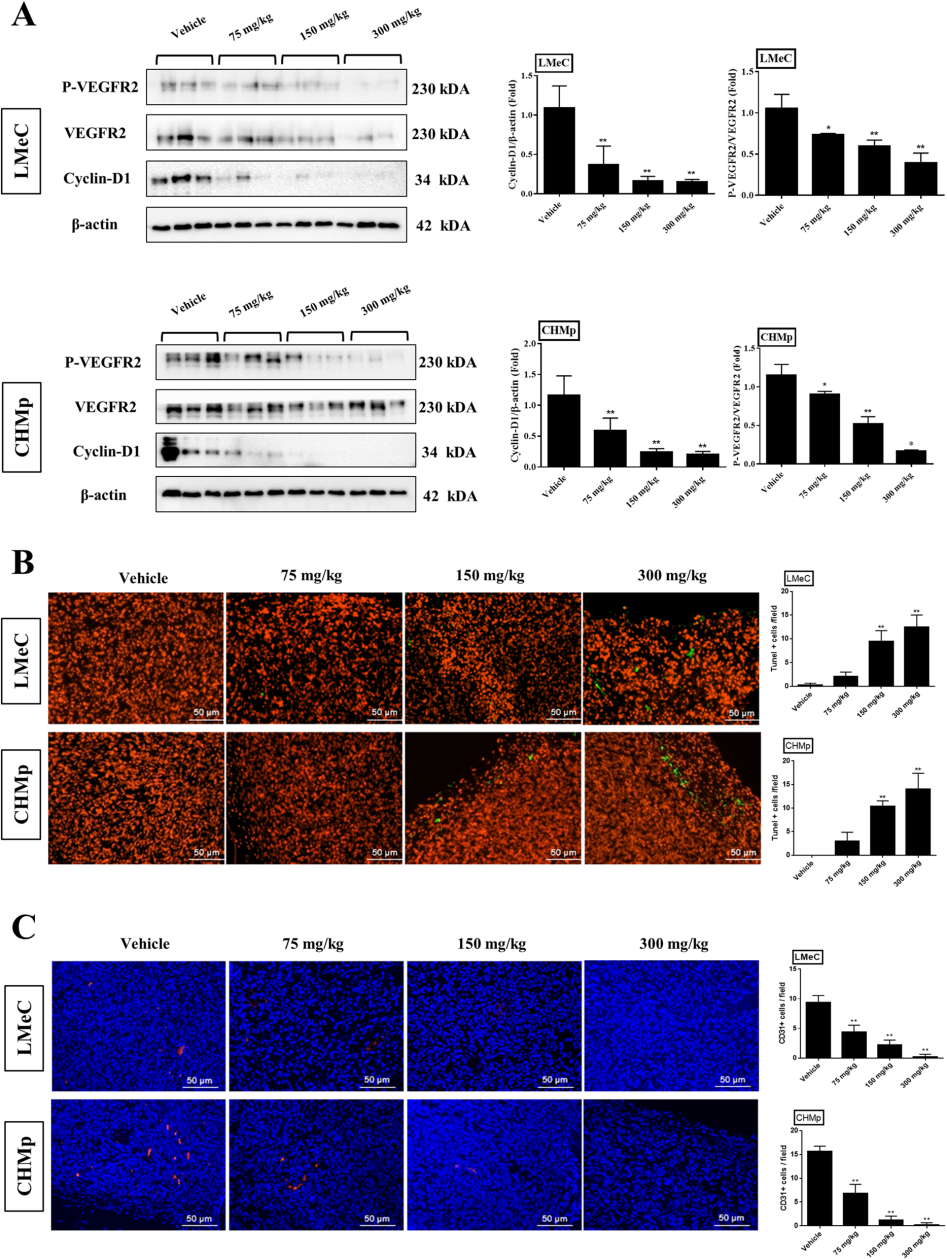
Effects of rivoceranib on tumour tissue in vivo. **A** The collected tumour tissues were subjected to western blot analysis for the evaluation of VEGFR2 activity and the expression levels of cyclin-D1. The intensity of the bands on the films was evaluated as the relative ratio of phosphorylated-VEGFR2 (P-VEGFR2) to the band intensity of VEGFR2 and the ratio of cyclin-D1 to the band intensity of β-actin. Cropped bands are displayed. Samples were derived from same experiment, and blots were processed in parallel. **B**, **C** TUNEL and anti-CD31 staining images of tumour sections in each group (vehicle and 75 mg/kg, 150 mg/kg, and 300 mg/kg rivoceranib-treated). TUNEL- and CD31-positive cells were counted in five random fields per group under a microscope. The data represent the mean ± standard deviation. * *p* < 0.05, ** *p* < 0.01 versus control group (vehicle group)

We further investigated the effects of rivoceranib on VEGFR2 phosphorylation and cyclin-D1 expression. The results revealed that cyclin-D1 expression and VEGFR2 phosphorylation were significantly decreased in the rivoceranib-treated groups in a dose-dependent manner (Fig. 6A). These results suggested that rivoceranib probably inhibited cyclin-D1 and downregulated VEGFR2 phosphorylation to reduce canine cell line viability in vivo.

### Rivoceranib induces cell apoptosis and inhibits angiogenesis in vivo

Five tumour tissues per group that were selected randomly were sectioned and stained with TUNEL and anti-CD31 to evaluate the effects of rivoceranib on cell apoptosis and angiogenesis, respectively. We found that rivoceranib treatment increased TUNEL-positive cells more than that observed with vehicle treatment in a dose-dependent manner in both cell lines (Fig. 6B). However, fewer CD31-positive cells were observed in the rivoceranib-treated tumour sections than in the control sections in both cell lines (Fig. 6C). Overall, these results indicated that rivoceranib showed apoptotic and anti-angiogenic activity in vivo.

## Discussion

Rivoceranib is a highly selective VEGFR2 inhibitor and is a second-generation anti-angiogenic drug that has been approved for the treatment of solid tumours in humans [13, 21, 22]. Several studies have also shown that VEGFR2 is highly expressed in canine malignant tumours [23–27]. In addition, Prado et al. and Inteeworn et al. attempted to evaluate the anti-tumour effects of sorafenib, a VEGFR inhibitor, in canine tumour cells [28, 29]. In this study, we evaluated the anti-tumour effects of rivoceranib in mouse xenograft models of canine melanoma and MGT.

Recent studies have demonstrated that rivoceranib is effective against diverse types of human cell lines such as hepatocarcinoma, non-small cell lung cancer, melanoma, and breast cancer [30–33]. Consistent with the results of previous studies, our results indicate that the cytotoxic and anti-tumour effects of rivoceranib can be attributed to the enhancement of apoptosis, cell cycle arrest, and inhibition of migration in canine tumour cell lines in vitro. We also demonstrated that the expression levels of cyclin-D1 which are well known for playing a key role in tumour growth and proliferation [34, 35] were significantly reduced by rivoceranib treatment in a dose-dependent manner in both cell lines. Although rivoceranib showed anti-proliferative effects against canine tumour cell lines (LMeC and CHMp) in vitro, it was difficult to evaluate whether these effects were associated with the VEGFR2 pathway because the canine tumour cell lines used in this study showed minimal expression of VEGFR2 in vitro (Supplementary Figure S1).

Therefore, we also determined the anti-tumour activity of rivoceranib in vivo with two xenograft mouse models of canine tumours: LMeC and CHMp. Interestingly, VEGFR2 was highly expressed in the tumours of xenograft mouse models containing LMeC and CHMp. After oral administration of rivoceranib at doses of 0, 75, 150, and 300 mg/kg in mice with LMeC or CHMp cells, the average tumour volume and weight were significantly reduced in a dose-dependent manner. Our western blot analysis results showed that rivoceranib significantly reduced the activity of VEGFR2 in vivo. Furthermore, evidence of the rivoceranib-induced increase in TUNEL-positive cells and fewer CD31-positive cells in treated tumour sections than in untreated tumour sections in both cell lines led us to hypothesise that rivoceranib reduced cell viability and tumour angiogenesis by inhibiting VEGF/VEGFR2 signalling.

The VEGFR family consists of VEGFR-1/Flt-1, VEGFR-2/KDR/Flk-1, and VEGFR-3/Flt-4 [33, 36, 37]. VEGFR2 exerts its biological function by coupling with VEGF cytokines to activate the VEGF/VEGFR2 signalling pathway, which is closely related to tumour angiogenesis and plays a crucial role in tumour cell adaptation to hostile environments [30, 38]. VEGF/VEGFR2 is well established to promote neighbouring vessel formation, thereby facilitating the delivery of nutrients for cancer cell survival, and previous reports have suggested that the expression of VEGFR2 correlates with poor prognosis [39, 40]. Rivoceranib is an oral anti-angiogenic drug that highly selectively inhibits VEGFR2 with a binding affinity 10 times higher than that of anti-angiogenic drugs sorafenib or vantalanib [14, 15].

There are some limitations in this study. First, we used a single cell line for canine melanoma and MGT. Second, as there are other anti-tumour underlying mechanisms, further studies using rivoceranib with other anti-tumour agents for synergic effects are needed. However, these results may provide a reference in veterinary medicine because this study was the first attempt to demonstrate the anti-tumour effects of rivoceranib in mouse xenograft models of canine tumours. Also, our data might provide scientific evidence for conducting clinical trials in canine patients.

## Conclusions

Our results showed that rivoceranib inhibits proliferation, migration, and cell cycle progression in two canine tumour cell lines (LMeC for melanoma, and CHMp for MGT) in vitro. In addition, we showed that rivoceranib inhibits tumour growth in xenograft mouse models through the enhancement of apoptosis and anti-angiogenic effects by inhibiting the VEGFR2 pathway. These results suggest that rivoceranib, a selective VEGFR2 inhibitor, might be a novel anti-angiogenic therapy for dogs with melanoma or MGTs.

## Methods

### Cell culture

The canine MGT cell line (CHMp) and the canine melanoma cell line (LMeC) was kindly provided by Professor Nobuo Sasaki [19, 20]. LMeC and CHMp cells were incubated in Roswell Park Memorial Institute-1640 medium and supplemented with 10% foetal bovine serum (FBS) and 100 U/mL of penicillin/streptomycin at 37 °C in a humidified atmosphere with 5% CO_2_. The medium was replaced every 2–3 days, and the cells were sub-cultured at 90% confluency.

### Cell proliferation assay

Rivoceranib powder was provided by HLB Life Science Co., Ltd. (Gangnam-gu, Republic of Korea) and stored at room temperature (20–25 °C). To determine the concentration of rivoceranib that affects canine cell line viability and proliferation, a cell proliferation assay was performed as described previously [18]. Briefly, a density of 1 × 10^3^ cells were plated onto 96-well cell culture plates (SPL Life Science, Pocheon, Korea) with 100 μL of culture medium containing rivoceranib (0, 3.125, 6.25, 12.5, 25, 50, or 100 μM). After culturing for 24, 48, and 72 h, the cell number was determined using the D-plus^tm^ CCK-8 assay (Dong-in Biotech, Seoul, Korea) according to the manufacturer’s instructions. Wells containing culture medium or 0.5% dimethyl sulfoxide with culture medium were used as the control group.

### Cell cycle assay

For cell cycle assay, as described previously [41], LMeC and CHMp cells were cultured in 6-well cell culture plates, allowed to adhere, and further cultured with increasing concentrations of rivoceranib (0, 12.5, and 25 μM). After 48 h, 1 × 10^5^ cells were collected, washed with cold PBS, and fixed with 70% cold alcohol at -20 °C for 2 h. The cells were then collected, washed again with PBS, and incubated with 500 μL of PI/RNase buffer (BD Biosciences, San Diego, CA, USA) for 30 min at room temperature. The samples were analysed using flow cytometry FACS (AriaII flow cytometer; BD Bioscience, San Jose, CA, USA).

### Wound-healing assay

The migration abilities of the cell lines were determined using a wound-healing assay as described previously [18]. Briefly, the cells were seeded in a 12-well culture plate at a density of 1 × 10^5^ cells/well. After reaching 100% confluence, the cells were induced using 2 μg/mL of mitomycin (EnzoLife Science, Farmingdale, NY, USA) for 2 h. The cells were manually scratched using a sterile 1000-μL pipette tip, and suspended cells were removed using phosphate-buffered saline (PBS). The various groups of adherent cells were cultured in serum-reduced complete medium (2% FBS) with rivoceranib (0, 12.5, and 25 μM). Cell migration was observed and captured under a microscope using the T Capture program (Tucsen Photonics, Fuzhou, China) at 0, 12, and 24 h. The migration ability was calculated relative to the gap size of the control group at 0 h.

### Apoptosis analysis

LMeC and CHMp cells were seeded in 6-well cell culture plates at a density of 1 × 10^5^ cells, allowed to adhere, and treated with different concentrations of rivoceranib (0, 12.5, and 25 μM) for 48 h. The cells were then dual-stained with annexin V and PI using the Annexin V Apoptosis Detection Kit I (BD Pharmingen, San Diego, CA, USA) as described previously [18]. The apoptosis rate of CHMp cells was analysed using a FACS cytometer (BD Bioscience, San Diego, CA, USA).

### Mouse xenograft model

Female NOD.CB17-Prkdc^SCID^/Acr mice aged 6 weeks and weighing 18–22 g were purchased from Raon Bio (Yongin, Korea). All mice were housed in a specific pathogen-free standard room under controlled temperature (19–23 °C) and humidity (50 ± 20%) conditions and a 12-h light–dark cycle. The study and all experimental procedures involving animals were approved by the Institutional Animal Care and Use Committee of KPC (P194007), and the experiments were performed in compliance with the guidelines for animal experiments. The study was also carried out in compliance with the ARRIVE guidelines. To induce tumours in the mice, 5 × 10^6^ CHMp cells or 1 × 10^7^ LMeC cells were suspended in 100 μL of PBS, mixed with 100 μL of Corning® Matrigel® Matrix (1:1 dilution, Corning Inc., New York, NY, USA), and then injected subcutaneously into the right flank of each mouse, as described previously [5]. After the predetermined inoculation and treatment periods, tumour dimensions were measured, and the volume was calculated using the following formula: 0.5 × width^2^ × length. The weight and tumour volume of the mice were monitored every 3–4 days. Mice were sacrificed on day 16 for the canine melanoma model and day 29 for the canine MGT model. Tissue samples of tumour were collected for further processing.

### Rivoceranib treatment

For rivoceranib administration, the reagent powder was dissolved in 0.5% carboxymethylcellulose (CMC; Sigma-Aldrich, St. Louis, MO, USA) solution. As described previously [42], when the tumours reached an average volume of 150–200 mm^3^, the mice were randomised into the following four groups (*n* = 10 each) and were orally treated as indicated: Group 1, 10 mL/kg 0.5% CMC solution (vehicle); and Groups 2, 3, and 4 were administered 75, 150, and 300 mg/kg rivoceranib, respectively, in 10 mL/kg of the vehicle. Rivoceranib and the vehicle were administered daily for 15 (CHMp) or 28 (LMeC) consecutive days, after which all mice were euthanized and tumour tissue samples were collected for further analysis.

### Western blot analysis

Total protein from the collected cell lines and tissue was extracted in PRO-PREP Protein Extraction Solution (Intron Biotechnology, Seongnam, Korea) on ice according to the manufacturer’s instructions. The protein concentration was measured using the Bio-Rad DC Protein Assay Kit (Bio-Rad, Hercules, CA, USA) as described previously [43]. The collected proteins (30 μg) were separated using sodium dodecyl sulphate gel electrophoresis, and the protein bands were transferred onto polyvinylidene difluoride membranes (EMD Millipore, Billerica, MA, USA). The membranes were incubated in 5% non-fat dry milk in Tris-buffered saline containing 0.1% Tween 20 for 1 h and incubated with antibodies against anti-cyclin-D1 (1:1000; LSBio, Seattle, WA, USA) and anti-phospho-VEGFR2 (1:1000; Cell Signaling Technology, Beverly, MA, USA) at 4 °C overnight. The membranes were incubated with anti-mouse or anti-rabbit immunoglobulin G (Santa Cruz Biotechnology, Dallas, Texas, USA) as the secondary antibodies (1:2000) for 1 h. Immunoreactive bands were normalised to β-actin (1:1000; Santa Cruz) or VEGFR2 (1:1000; Cell Signaling Technology) and visualised using SuperSignal West Pico PLUS Chemiluminescent substrate (Advansta, Menlo Park, CA, USA).

### Immunofluorescence analysis

Xenograft tumour tissues obtained from mice were fixed in 10% formalin, and paraffin-embedded 4-μm-thick tissue sections were deparaffinised in xylene and rehydrated with ethanol. The tumour apoptosis rates were determined based on TUNEL staining (Apo-BrdU DNA Fragmentation Assay Kit; BioVision, San Francisco, USA), as described previously [5]. For the analysis of angiogenesis ability in the tumour tissue, the slides were incubated overnight at 4 °C with antibodies against CD31 (1:100; Thermo Fisher Scientific, Waltham, MA, USA). After washing three times, the tumour sections were incubated with the secondary antibody (1:200; sc-516251; Santa Cruz Biotechnology) for 1 h at room temperature (protected from light). Finally, the slides were mounted in Vectashield mounting medium containing 4,6-diamidino-2-phenylindole (Vector Laboratories, Burlingame, CA, USA). The immunoreactive cells were counted in six random fields per group using an EVOS FL microscope (Life Technologies, Darmstadt, Germany).

### Statistical analysis

Data are shown as mean ± standard error. Differences between the groups were compared by one-way analysis of variance or Student’s t-test using GraphPad Prism v.6.01 software (GraphPad Inc., La Jolla, CA, USA). A value of *P* < 0.05 was considered statistically significant.

## Supplementary Information

**Additional file 1: Supplementary Figure S1.** Western blot analysis of total and phosphorylated VEGFR2 in tumour cell lines (LMec and CHMp). The expression levels of total and phosphorylated VEGFR2 are weak for in vitro experiments. Cropped bands are displayed. Samples were derived from same experiment, and blots were processed in parallel.

**Additional file 2: Supplementary Figure S2.** The full-length blots with specific protein bands used in this study. (A) The full-length blots used in Fig. 1B. (B,C) The full-length blots used in Fig. 6A.

## Data Availability

The data that support the findings of this study are available from the corresponding author upon reasonable request.

## Abbreviations

MGT: Mammary gland tumour
VEGF: Vascular endothelial growth factor
VEGFRs: Vascular endothelial growth factor receptors
FACS: Fluorescence-activated cell sorting
FBS: Foetal bovine serum
CCK: Cell counting kit
PBS: Phosphate-buffered saline
