# Supplementary figures and images for: Anti-tumor effects of rivoceranib against canine melanoma and mammary gland tumour in vitro and in vivo mouse xenograft models
Source: BMC Vet Res. 2021 Oct 26;17:338. doi: 10.1186/s12917-021-03026-1 (PMC8546947; doi:10.1186/s12917-021-03026-1)

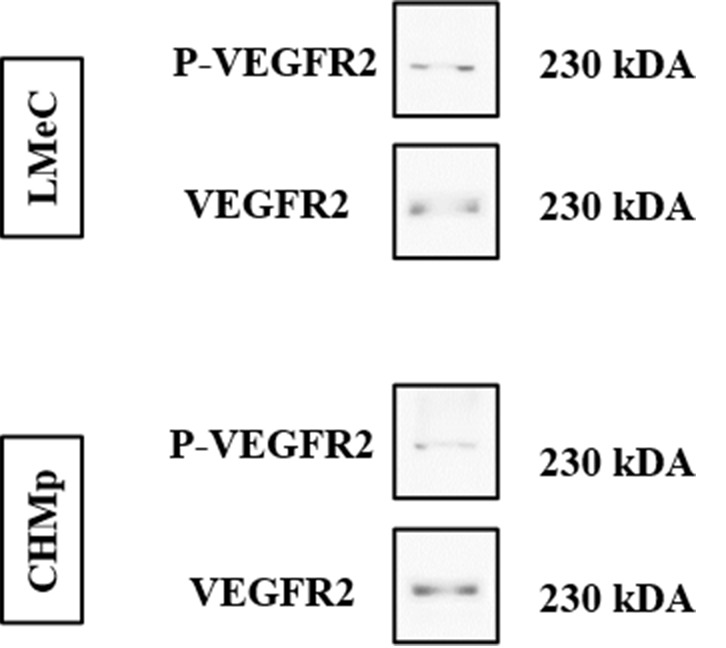

Supplement: Supplementary file 1 — Additional file 1: Supplementary Figure S1. Western blot analysis of total and phosphorylated VEGFR2 in tumour cell lines (LMec and CHMp). The expression levels of total and phosphorylated VEGFR2 are weak for in vitro experiments. Cropped bands are displayed. Samples were derived from same experiment, and blots were processed in parallel. [file 12917_2021_3026_MOESM1_ESM.tif]

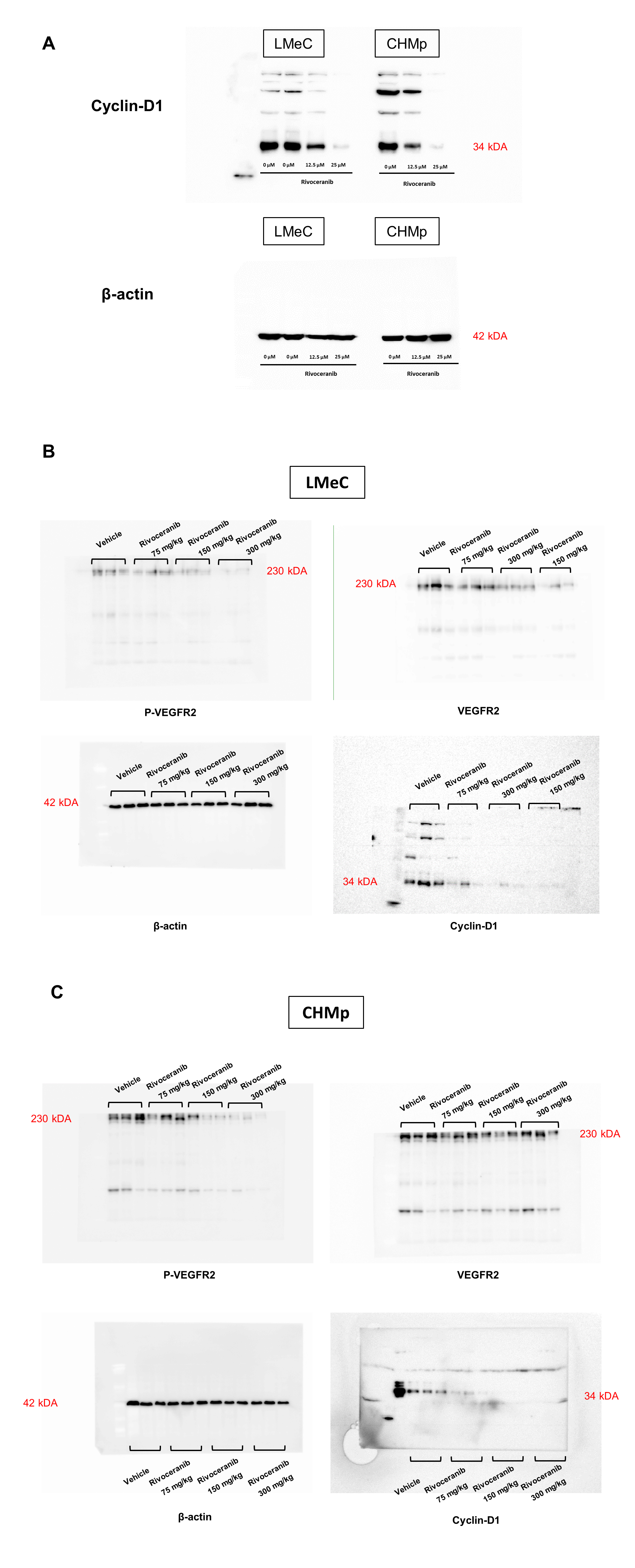

Supplement: Supplementary file 2 — Additional file 2: Supplementary Figure S2. The full-length blots with specific protein bands used in this study. (A) The full-length blots used in Fig. 1B. (B,C) The full-length blots used in Fig. 6A. [file 12917_2021_3026_MOESM2_ESM.tif]
